# Suitability of Automatic Photogrammetric Reconstruction Configurations for Small Archaeological Remains

**DOI:** 10.3390/s20102936

**Published:** 2020-05-22

**Authors:** Manuel Rodríguez-Martín, Pablo Rodríguez-Gonzálvez

**Affiliations:** 1 Department of Mechanical Engineering, Universidad de Salamanca, Avenida Fernando Ballesteros, 0, Béjar, 37700 Salamanca, Spain; ingmanuel@usal.es; 2 Department of Technology, Universidad Católica de Ávila, C/ Canteros s/n, 05005 Ávila, Spain; 3 Department of Mining Technology, Topography and Structures, Universidad de León, Avenida Astorga, s/n, 24401 Ponferrada, Spain

**Keywords:** macro-photogrammetry, 3D reconstruction, archeology, cultural heritage, documentation, digital preservation, point cloud

## Abstract

Three-dimensional (3D) reconstruction is a useful technique for the documentation, characterization, and evaluation of small archeological objects. In this research, a comparison among different photogrammetric setups that use different lenses (macro and standard zoom) and dense point cloud generation calibration processes for real specific objects of archaeological interest with different textures, geometries, and materials is raised using an automated data collection. The data acquisition protocol is carried out from a platform with control points referenced with a metrology absolute arm to accurately define a common spatial reference system. The photogrammetric reconstruction is performed considering a camera pre-calibration as well as a self-calibration. The latter is common for most data acquisition situations in archaeology. The results for the different lenses and calibration processes are compared based on a robust statistical analysis, which entails the estimation of both standard Gaussian and non-parametric estimators, to assess the accuracy potential of different configurations. As a result, 95% of the reconstructed points show geometric discrepancies lower than 0.85 mm for the most unfavorable case and less than 0.35 mm for the other cases.

## 1. Introduction

Geotechnologies encompass different sensors and computer algorithms for the acquisition, modeling, and/or analysis of spatial features [1]. Moreover, different geotechnologies are available to document, model, and analyze small objects. Recent advances in geotechnologies has enabled the use of a wide range of sensors that record, catalog, and study cultural heritage sites [2,3,4]. Some of these geotechnologies include laser scanning, structured light systems, and photogrammetry. In recent years, these techniques have demonstrated value to visual inspection [5]. The generation of three-dimensional (3D) digital models of heritage assets such as monuments or excavations is an important task in areas such as heritage documentation [6]; inspection, and restoration [7]; project planning and management [8]; virtual and augmented reality [9]; and other areas of scientific research [10].

Heritage is an important cultural, social, and economic resource that enriches societies who appreciates and know how to maintain a site’s authenticity, integrity, and/or the memory of its original state, as well as the probable evolution to its current state. A high accuracy 3D model of a heritage element is of great value for documenting, evaluating, analyzing, and monitoring the heritage. Its physical properties [11,12], and its virtualization. Moreover, a 3D model acts as a base to reestablish missing elements in a reconstruction of a heritage element’s current remains, which achieves a complete virtual 3D reconstruction of the element [13]. Based on geometry modelling applications (e.g., Maya, 3D Studio Mark), we can generate virtual models similar to real ones. However, these applications require considerable learning [10] and working time. Furthermore, while using these applications it is not possible to reconstruct a heritage artifact with total geometric and chromatic fidelity.

The documentation of heritage models as 3D models can be done with different techniques such as laser scanning with portable mobile mapping systems (PMMS), static laser systems [6], structured light systems [14], and photogrammetry [15]. For small size objects, only three techniques can be used for an accurate three-dimensional reconstruction: laser scanning, structured light system (white, blue, or infrared (IR) light), and photogrammetry. This is true even for industrial tasks such as non-invasive quality control and documentation [16,17,18].

Laser scanning is a technique based on the use of a controlled light source (active technique) to sweep the object’s surface and analyze the reflected energy [17]. This type of system has a price between 80–100 times the cost of a basic photogrammetric equipment to achieve a submillimeter resolution [19]. Structured light-based depth cameras project specific light patterns that extract the geometric information of the scene based on the structured-light triangulation principle [20]. These systems are versatile and provide good results, but the reflections generate zones without information in the model, which can create problems in the 3D documentation process [18]. In combination with Structure for Motion (SfM) techniques, photogrammetry has been developed in recent years and is an attractive alternative to laser scanning systems [21,22] and structured light systems. The input for this process is the collection of single images acquired using an off-the-shelf camera, which can be even equipped in platforms such as drones [23]. In recent years, the image-based modelling strategy (SfM) has positioned itself as an attractive alternative to active scanning systems. On the one hand, it is flexible; it can be integrated into different types of platforms (e.g., drones [24]) and employed to document a wide range of scenarios and objects [25]. On the other hand, it is low-cost because the necessary hardware is a standard photographic camera and lens. It is also worth highlighting that the features of the generated dense point cloud (in terms of radiometric information provided, high spatial density, and precision) place SfM at a vantage position in the evaluation of heritage buildings and elements by integrating advantages of computer vision (automation and flexibility) and photogrammetry (accuracy and reliability) [26] to obtain high density 3D models whose accuracy can compete with laser scanner systems [27,28].

The main weakness of this technique is the dependence of a specialized camera operator who can configure the camera parameters and obtain images in the right way (e.g., properly focused, without blurring, with proper exposition, low noise level, etc.). If the images are not acquired adequately, the subsequent 3D reconstruction will be affected by the presence of significant noise and/or reconstruction errors. Moreover, the positioning of the control points and their marking in the image are also critical steps to assure the accurate reconstruction with metric units. The latter is significant for the assembly of dismantled heritage elements and/or missing parts [29]. To obtain the complete geometry of an object (360° image acquisition), it is necessary to take shots around it according to a specific path or, alternatively, keep the camera fixed and rotate the object at predefined angle steps [30,31]. Nevertheless, this last approach implies higher preprocessing times since the background has to be removed from the images so that it does not take part in the reconstruction process. However, the main challenges include optimizing the number of images to avoid excessive processing times, stabilizing the camera due to long exposure times in low-light conditions, considering the presence of hard reflections due to direct light sources, and, finally, establishing the camera-object distance as a constant. The latter is of special significance for very small objects and/or very high spatial resolutions due to the limited depth of field of macro lenses [32,33]. Including a reference element in the scene that remains static during the capture process can be a cumbersome task in some cases and can even hide details of the piece itself.

Different free packages are available for the generation of photogrammetric models such as GRAPHOS [34], MICMAC [35], Regard3D [36], and ColMAP [37]. Nevertheless, these applications could be difficult to use for researchers and professionals in the cultural heritage field, especially for those who are not experts in photogrammetry. A comparison of some open-access software such as Metashape [38] takes into account applications for mesh generation, 3D sharing, and visualization tools. Metashape [39] is one of the most used commercial photogrammetric and SfM packages. It has been used as ground through for comparatives with respect to other applications [10,38]. In this case, Metashape software is taken as reference for this experimentation due to its popularity among non-experts in photogrammetry, conservation, and cultural heritage documentation.

Finally, it should be noted that in the Mosul project, crowd-sourced photogrammetry was proposed as an opportunity to visualize and document lost heritage using images with unknown parameters taken without photogrammetric knowledge [40,41]. Such occurrences are an example of the scientific community’s interest in extending the photogrammetric process to non-expert users to preserve and document cultural heritage.

This article aims to provide an automatic workflow for image acquisition with a commercial digital single lens reflex (DSLR) camera. We apply a robust statistical comparison methodology to real small size archaeological pieces obtained with two different lenses and with different calibration processes (pre-calibration and self-calibration). Herein, this article advises non-experts in photogrammetry and heritage specialists working in data acquisition and small archaeological artifact modelling.

## 2. Materials and Methods

**Figure 1:**
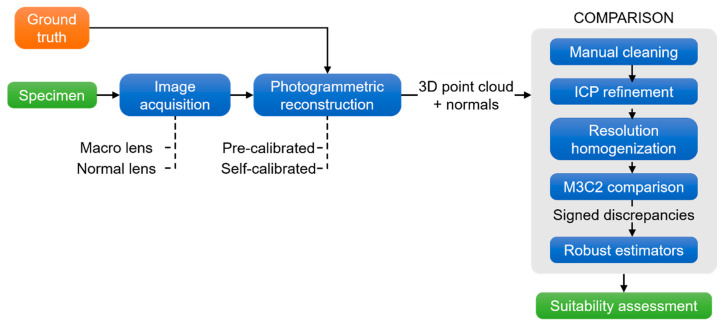
Workflow of the proposed comparison methodology. ICP: Iterative Closest Point; M3C2: Multiscale Model to Model Cloud Comparison; 3D: Three-Dimensional.

In this section, the specimens employed for the case study and the evaluation are described. The methodology is structured in three phases: the dense point cloud generation of the archaeological specimens, the 3D signed comparison, and the evaluation that employed robust statistical estimators. The complete methodology is summed up in the Figure 1.

### 2.1. Materials

In the present section, firstly, the archaeological specimens selected to test the photogrammetric configurations are described. Secondly, the different photogrammetric devices and their technical specifications are presented. Finally, we describe the process based on the articulated coordinate measurement machine employed to define the ground truth.

#### 2.1.1. Archaeological Specimens

**Figure 2:**
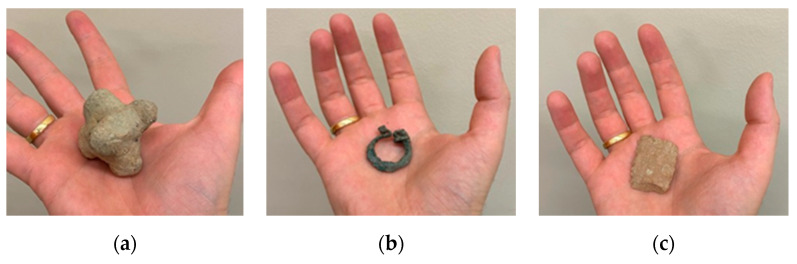
Archaeological specimens: (**a**) separator; (**b**) brooch; and (**c**) racloir.

For this study, three small archaeological objects with different shapes and degrees of complexity (Figure 2) were chosen to cover the majority part of documentation situations. Firstly, a baked clay separator presents a regular volume and a three axes symmetry. This geometry is closer to a cubical one and was expected to be the easiest to reconstruct. Secondly, the copper brooch was closer to a toroidal geometry. The small size of the ring shanks and both ring heads would be prone to cause reconstruction noise and occlusions, therefore it is a challenging piece. Finally, the third specimen is a silex racloir characterized by a flatter and smother geometry than the rest. However, the sharp edge is expected to be problematic during the reconstruction phase due to the difficulty of finding and matching key points.

In Table 1, the main characteristics of the three specimens is briefly described.

#### 2.1.2. Photogrammetric Equipment

For the photogrammetric reconstruction, a conventional DSLR camera was employed (Canon 77D, Canon Inc., Tokyo, Japan) with two different lenses: a Canon EF-S 60 mm macro-lens (hereinafter referred to as ‘macro’) and a conventical zoom lens Canon EF-S 18–55 mm (referred to as ‘zoom’) (Table 2). Since the camera and lens are commercial and semiprofessional, their cost is more affordable than professional DSLR equipment. This type of camera is widely used by archaeologists and experts because both the sensor and the features are sufficiently adequate to perform most tasks related to the documentation and evaluation of cultural heritage sites.

**Figure 3:**
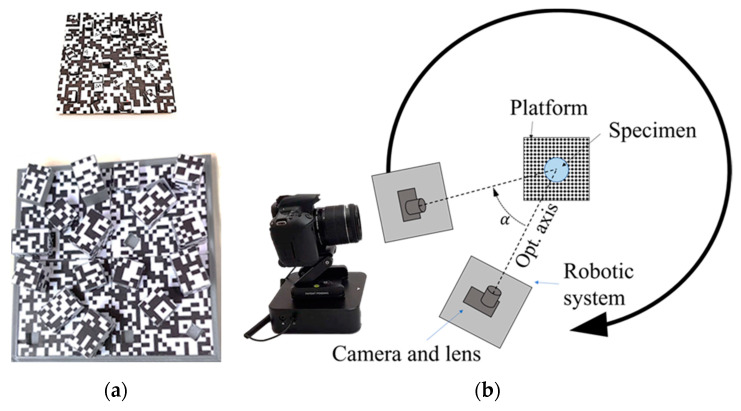
Photogrammetric equipment and data acquisition protocol: (**a**) calibration pattern for macro lens (up) and for zoom lens (down); (**b**) robotic device linked with the DSLR camera and automatic movement of the platform through the configured path (without operator).

To automate image data acquisition and avoid reconstruction biases due to the differences related to the camera’s external orientation, the camera and lens are attached to the robotic device that allows for the control of the camera path and the image recording (Figure 3b). For the present study, Edelkrone’s robotic system DollyOne was chosen [42] which allowed us to create linear or curved camera paths on flat surfaces. The device is controlled using the manufacturer’s app, which allowed for the final user to set up the path, the camera’s position on the road, and the number of acquired images.

In order to provide the metric scale to all specimens reconstructed by the aforementioned configuration, a calibrated rectangular platform was designed ad-hoc. The specimen will be placed on the platform, which acted as a rotation center for the circular path of the robotic system. The platform was characterized by the presence of 18 georeferenced control points in the base (six coplanar points) and on the edges (12 points being coplanar three of them for each lateral face). The coordinates of these ground control points (GCP) were provided by an articulated coordinate measurement machine (ACMM) with an expected accuracy better than 0.1 mm. Additionally, a pseudo-random texture pattern was included on all the surfaces of the platform to increase the number of the image key points. Therefore, there was an ease of use for the camera orientation phase with regard to 3D reconstruction.

The robotic device and the calibrated platform were located into a white box to avoid the reflects from direct light sources. Additionally, a tripod-mounted spotlight (55 W and 5500 K) was located just above the lightbox (1.20 × 1.20 × 1.20 m) to provide proper illumination to the scene (inside of the box) in a diffuse way (Figure 3b). By this configuration, the reconstruction uncertainty generated by the light sources was highly reduced.

Finally, for the generation of the dense 3D point clouds, the software Metashape [39] was employed. This solution allowed us to cover the whole reconstruction process and to introduce the coordinates of the GCP.

#### 2.1.3. Articulated Coordinate Measurement Machine

**Figure 4:**
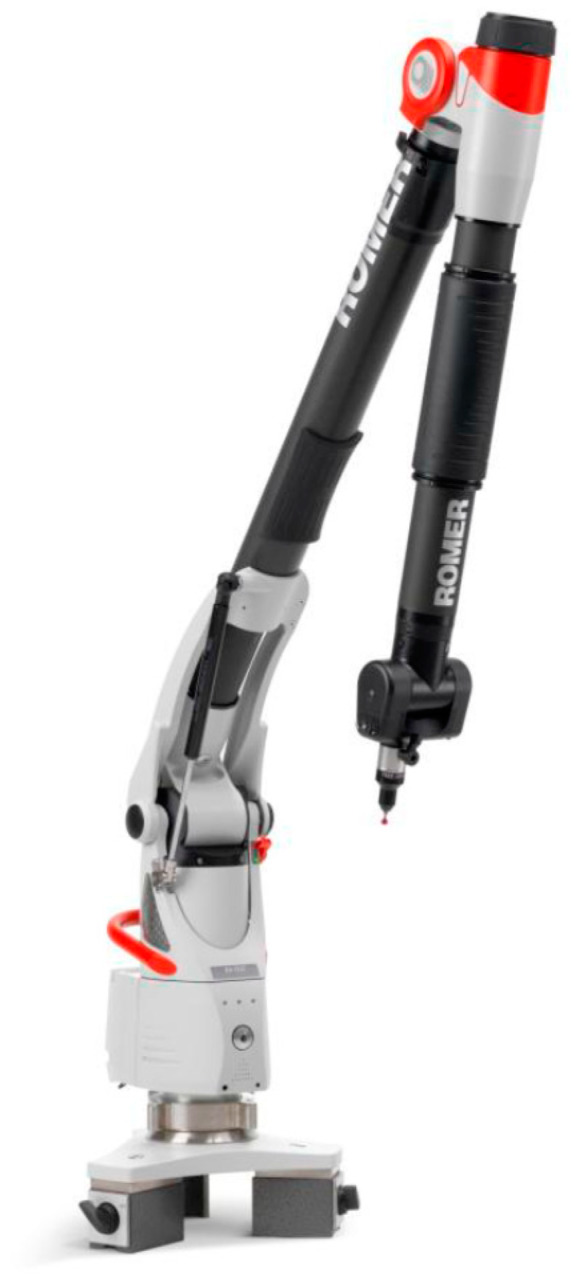
ACMM Hexagon Metrology Absolute Arm 7325SI employed for the ground truth. Image courtesy of Hexagon Manufacturing Intelligence division.

For the definition of the external reference system, an ACCM (Hexagon Metrology Absolute Arm 7325SI, Hexagon Metrology S.A., Cerdanyola del Vallés, Spain) was employed to probe the GCP, which is common to all data acquisition (Figure 4). This instrument, also known as the coordinate measurement machine, is based on the contact measurement through the probe testing, which defines the ground truth with the highest precision allowable by the instrument. The probe of the ACCM is used to extract the control points coordinates located on the platform. The main technical specifications of this metrological tool are shown in Table 3.

As shown in Figure 1 and Table 3, the ACMM system played the ground truth role because of contact measurements or probing was the most precise data acquisition since the measurement was carried out by physical contact. Although this measurement technique encloses limitations (e.g., working range or the type of object where can be applied), the contact measurement can be guided by a human operator or an industrial machine along the object. When both (probe and surface) were in contact, the 3D coordinates of the object were recorded. The points’ coordinates were measured using ACMM and were saved. They remained constant for the calibrated platform, unless deformed or deteriorated. Therefore, the final non-expert users will not need to recalibrate it again or use an ACMM.

### 2.2. Methodology

Herein, we present the 3D reconstruction pipeline with the protocol to assess the suitability of different photogrammetric configurations.

#### 2.2.1. 3D Reconstruction

Firstly, the robotic system with the attached camera was oriented towards the object. Subsequently, the photographic settings were established to ensure an adequate exposition of the object in relation to the scene light. Thus, the robotic device connected to the camera was programmed to automatically take convergent shots around the specimen without a human operator (Figure 3b). The robotic device and the camera rotated (*α*) around the referenced platform inside the light box, which always remained closed to maintain proper light distribution: the robotic system automatically moved, stopped, took the image, and moved again.

Once data was acquired, the photogrammetric processing protocol was applied. A circumference detection algorithm was applied to obtain the position of the center of the circumference [17]. The *x, y,* and *z* coordinates measured with ACMM were assigned to the centers of the circumferences as control points used as external reference frames for keeping the same special reference system for all point clouds generated during the research. In this way, they were compared.

Initial camera calibration parameters (pre-calibration) were obtained using two different calibration patterns designed ad-hoc and manufactured for this research, one of them for each lens due to the different field of view (Figure 3a). When these parameters were established, the photogrammetric reconstruction process was implemented using Metashape [39] and coordinates of the control points are included in the pipeline as GCPs. Image matching was carried out on the original images, whereas the densification was carried out with the original photos downscaling by a factor of 4, while the built-in filtering algorithms were set at low values in order to distinguish the small details of the specimens.

The described process was applied for the two different lenses (Table 2), as well as for a previous known initial calibration (pre-calibration), and self-calibration process, keeping all the other aforementioned parameters unchanged.

#### 2.2.2. Suitability Assessment

To assess the suitability and precision of each of the tested configurations, the resulting 3D photogrammetric models were compared based on signed discrepancy values using distances measured along the computed normal vector. This comparison was carried out using Cloud Compare software [43].

In the accuracy assessment of data provided by photogrammetry, the hypothesis that errors follow a Gaussian distribution was hardly verified [28,44,45]. This behavior might be caused by the presence of residual system errors but also unwanted objects not correctly filtered out from the data. Therefore, the possible presence of systematisms and/or outliers hindered the use of Gaussian statistics like the mean and standard deviation, since they did not provide a suitable analysis [46]. For this reason, the following robust estimators were adopted in the present study: the median *(m)*, the normalized median absolute deviation (NMAD) (1), the square root of the biweight midvariance (BWMV) (2), and the interpercentile ranges (IPR).

(1) NMAD =1.4826⋅MAD

(2) $$\mathrm{BWMV}=\frac{n\sum_{i=1}^{n}a_{i}{\left(x_{i}-m\right)}^{2}{\left(1-U_{i}^{2}\right)}^{4}}{{\left(\sum_{i=1}^{n}a_{i}\left(1-U_{i}^{2}\right)\left(1-5U_{i}^{2}\right)\right)}^{2}}$$

(3) $$a_{i}=\{\begin{array}{c} 1,if\left|U_{i}\right|<1\\ 0,if\left|U_{i}\right|\geq 1 \end{array}$$

(4) $$U=\frac{x_{i}-m}{9\mathrm{MAD}}$$

The above equations represent the median absolute deviation (MAD) (5), i.e., the median (*m*) of the absolute deviations from the data’s median (*m_x_*):

(5) $$\mathrm{MAD}=m\left(\left|x_{i}-m_{x}\right|\right)$$

Please note that for asymmetric distribution, it was not possible to provide a plus-minus range, therefore an absolute inter-percentile range at multiple confidence intervals was provided (50%, also known as interquartile range, 90% and 99%), and additionally some percentile values such as 2.5%, 25%, 75%, and 97.5%).

The hypothesis that errors follow a Gaussian distribution was checked according to graphical methods such as the quantile-quantile (Q-Q) plot [47], which is well-suited for very large samples [28]. The Q-Q plot depicts the quantiles of the empirical distribution plotted against the theoretical quantiles of normal distribution. If the actual distribution is normal, the Q-Q plot should provide a straight line. A big deviation from the straight line indicates that the distribution of the errors is not normal. If the samples are not normally distributed, either due to the presence of outliers or because of a different population’s hypothesis, a robust model based on non-parametric estimation should be employed.

3D points’ discrepancies were computed in consonance with Multiscale Model to Model Cloud Comparison (M3C2) [48], which performed a direct comparison of the 3D point clouds and avoided the preliminary meshing phase. The algorithm was divided into two sequential steps: estimation of normal vectors and distance computation. As a result of the photogrammetric processing, the 3D point cloud was computed with normal vectors and therefore the point cloud’s normal vectors were used to extract the local distance between the two clouds. Due to the high number of points (several millions) not all of them were employed as core points for the computation, but only the subsampled.

The discrepancies were associated to every 3D photogrammetric point and could also be assessed not only numerically by the central tendency and dispersion but visually (discrepancy map), identifying any kind of systematic pattern. The robust statistical estimators were computed by a custom script as well as the in-house statistical software (STAR: Statistics Tests for Analyzing of Residuals) [49].

Since a direct comparison would report the intrinsic 3D model discrepancies plus the external referencing errors, the photogrammetric models’ alignment was refined based on the iterative closest point (ICP) [50] algorithm to assess its precision. The a-priori result was a normal error distribution where any departures from it was associated with in-model systematic errors.

## 3. Results

Firstly, different empirical pre-tests were carried out in situ to obtain optimal camera parameters in relation to scene illumination and limited depth of field of the macro lens. The focal length of the zoom lens was fixed at 35 mm as a compromise between image definition and field of view, considering the space available inside the light. ISO sensibility was set at ISO-100 to reduce sensor noise as much as possible, which could have affected the photogrammetric process. The aperture was established at f/14 for all experiments to achieve an adequate depth of field (especially for the macro lens) without excessively affecting the exposition of the scene. The shutter speed was set to automatic, since the robotic device stopped at every position and avoided camera vibrations. The external reference frame was established by four reference points distributed on the base and on the edges of the platform whose coordinates were integrated in the photogrammetric process.

**Figure 5:**
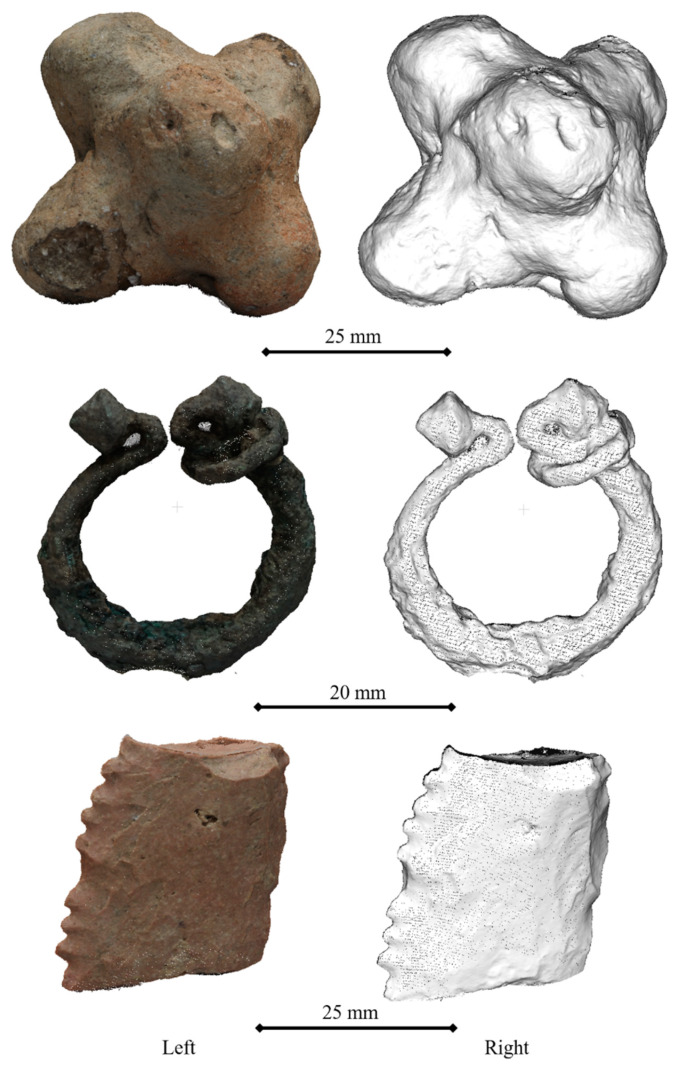
Dense 3D point clouds with texture (**left**) and without texture (**right**), generated using a pre-calibrated zoom lens.

**Figure 6:**
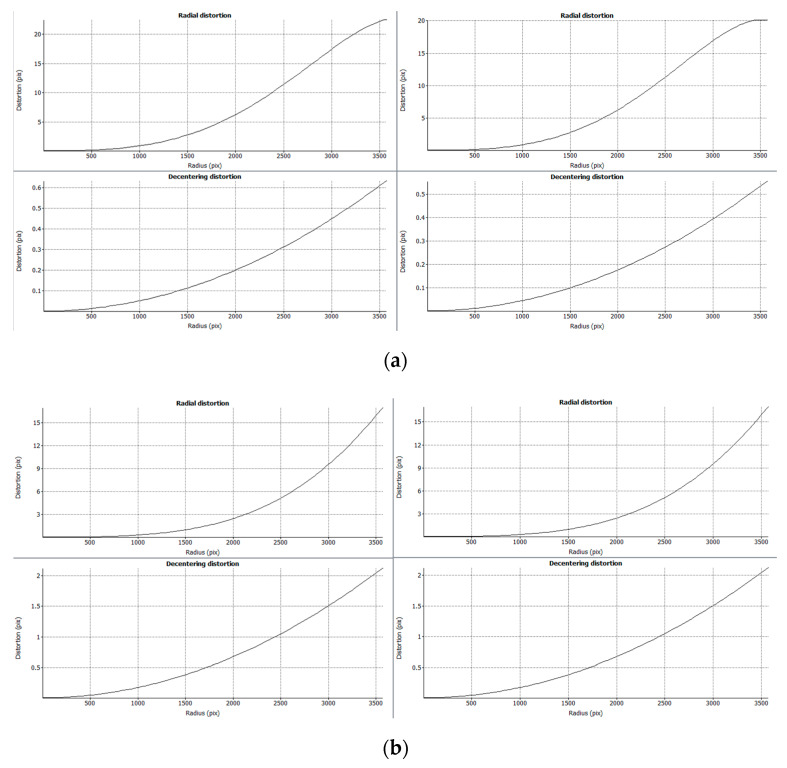
Examples of calibration curves for the specimen 1 (separator): (**a**) zoom lens pre-calibrated (left) and self-calibrated (right); (**b**) macro lens pre-calibrated (left) and self-calibrated (right).

The photogrammetric reconstruction was performed following the steps described in Section 2.2.1. In this way, four different point clouds were obtained for each specimen, one for each lens, and one for each camera calibration process (pre-calibrated vs. self-calibrated). An example of dense point clouds for each specimen (geometry and texture) is shown in Figure 5. During the calibration process, a radial and decentered distortion curve were obtained (Figure 6).

The distortion curves of the zoom lens (Figure 6a) show differences in the last one-third of the diagonal, whereas for the macro lens (Figure 6b) there are no significant differences. One of the aims of this research was to evaluate if these differences significantly impact the reconstruction process. The initial hypothesis was that, due to the higher field of view of the zoom lens, during the self-calibration the edges of the images would not contribute with key points for the camera orientation and internal parameter determination step. Yet, since reconstruction was carried out near to the center of the images, this difference was not relevant in terms of geometric discrepancies.

Additionally, in Table 4 and Table 5, the main summary of the reconstruction process is shown. In Table 4, the results of the bundle adjustment solution for each case are presented, using four GCPs as reference points to scale the model and the rest of available GCPs as check points. The accuracy reported by the check points was increased due to the oblique point of view of the GCPs distributed in the base. Please note that since photogrammetric models’ alignment was refined based on ICP (as stated in Section 2.2.2.) the error reported by the check points does not affect the subsequent analyses.

Table 5 lists the average point density achieved according to an ideal equilateral triangular distribution for a circular neighborhood [51]. It is shown that the zooms lens, for the same photogrammetric reconstruction parameters, achieved a lower spatial resolution. According to the focal length reaction (35 mm vs. 60 mm), the GSD of the zoom lens was approximately 71% higher than the macro lens. It was expected that the macro lens would achieve a resolution 2.9 times higher than the zoom lens. However, in Table 5, this relation is not achieved due to the different specimen shapes.

After carrying out the ICP refinement to dismiss any possible error due to movement of the archaeological sample in the base, a point density reduction was applied. Due to the high number of points of each sample (Table 5), in order to speed up the computation process, a spatial subsampling at 0.1 mm was carried out using the function incorporated in CloudCompare [43]. Additionally, a manual cleaning was applied to all point clouds to remove the points related to the base and reusable adhesive putty. The discrepancies were computed using the M3C2 algorithm [48] and exported to the obtention of the statistical estimators.

The above steps are applied for the next subsections: comparison between pre-calibration and self-calibration (Section 3.1) and comparison between the use of macro and zoom lenses (Section 3.2).

### 3.1. Calibration Comparison

To establish a range of confidentiality during the comparison between both photogrammetric point clouds (pre-calibrated vs. self-calibrated), a statistical analysis was carried out (Table 6 and Table 7). The Gaussian estimation was provided by the mean and the standard deviation. Regarding the robust estimation, the central tendency of the error was estimated by the median and the error dispersion as the square root of the biweight midvariance (2) and NMAD, or normalized MAD (1).

**Figure 7:**
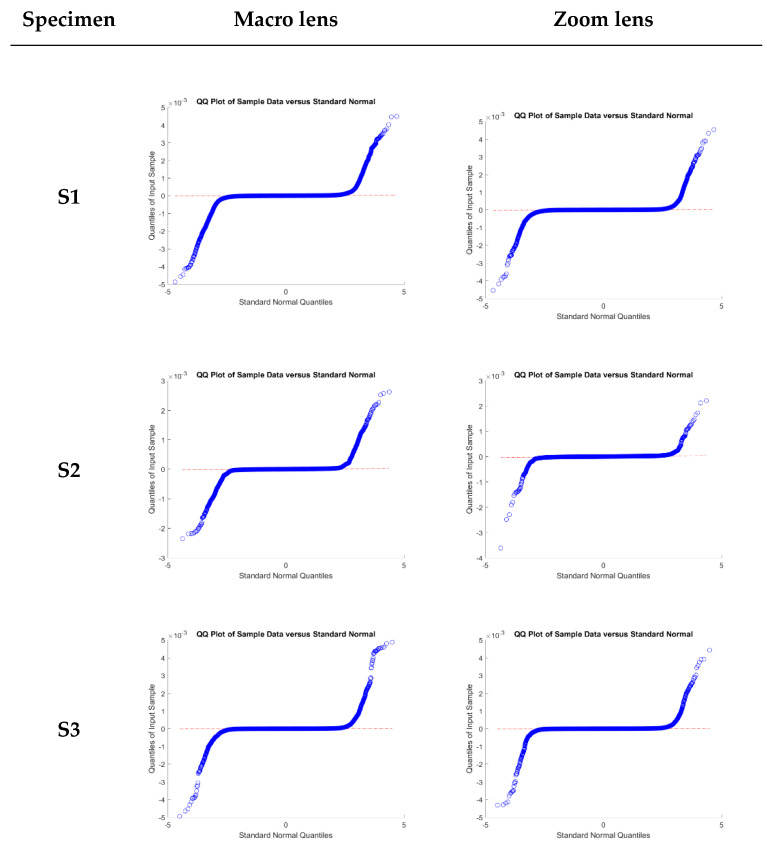
Quantile-quantile (Q-Q) plots of relative discrepancies between the different configurations and archaeological specimens.

As a global conclusion, there were no significant differences for all the tested configurations, being that the discrepancies in all cases compatible with zero. Please note that the overestimation of error, both for the macro and zoom lens, for the classical Gaussian approach and the normality condition was not met in any of the six tested cases, as illustrated in Figure 7, where the Q-Q plot to confirm the non-normality of the samples.

The absolute inter-percentile range at different confidence intervals (Table 7) provides an additional insight into the reconstruction differences for both approaches (pre-calibrated/self-calibrated). It can be noted that at a 95% confidence level (difference of the percentile 2.5% and 97.5%) is compatible with zero (a value lower than the ACMM precision).

Finally, in Table 7, the Gaussian confidence intervals overestimate the error range in all cases, stressing the importance of a normality assessment and the use of robust estimators for the geomatic products evaluated.

### 3.2. Lens Comparison

The comparison carried out in the previous subsection was repeated, but in this case for the pre-calibrated point clouds of the macro and zoom lenses. In Table 8, the median values were expected to be close to zero after the application of the ICP registration algorithm. A slightly higher value was appreciated for specimens 1 and 3, which points out some registration error or reconstruction deformation. Despite their small value (lower than 0.1 mm), they could be of relevance in the high detail reconstructions of small archaeological objects.

In Table 8, the robust dispersion values (NMAD and BWMV) are a good indicator of the precision degradation due to the use of a conventional zoom lens in relation to the macro lens best suited for small artifacts. In all cases, the dispersion value was almost ±0.1 mm, which matches the applied subsamples. As in Section 3.1, the Gaussian error dispersion was overestimated due to the asymmetrical shape of the discrepancy distribution, highlighting the importance of an adequate statistical parameter election.

**Figure 8:**
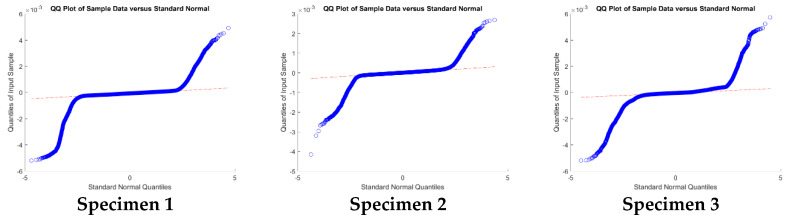
Q-Q plots of relative discrepancies between the macro and zoom lenses reconstruction for the separator (specimen 1), the brooch (specimen 2), and the racloir (specimen 3).

The obtained values were analyzed with a Q-Q plot to confirm the non-normality of the sample (Figure 8). This fact was hinted by the percentile values and the skewness and kurtosis parameters (these two are not listed in the table). The samples do not follow a normal distribution (Figure 8); it is not possible to infer the central tendency and dispersion of the population according to Gaussian statistics parameters like the mean and standard deviation. For that reason, the accuracy assessment was carried out based on robust alternatives, using non-parametric assumptions such as the median value and the square root of the biweight midvariance (2) (Table 8).

Moreover, the robust estimators provide a clearer view of the error distribution, as for example the absolute inter-percentile range at different confidence intervals (Table 9).

**Figure 9:**
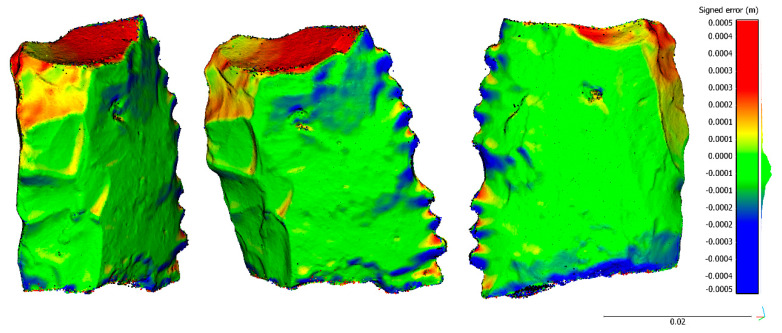
Different views of the spatial distribution of discrepancies between zoom lens reconstruction versus macro lenses for the specimen S3 (racloir): front view (**left**); diagonal front-right side view (**center**); and back view (**right**).

The difference of the percentile, 2.5% and 97.5%, (95% confidence level) is approximately 0.35 mm for the two cases, and 0.84 mm for the S3 case (racloir). The high error of the last specimen (S3-racloir) was caused by the top part of the sample (Figure 9), which was a weak area due to the camera configuration. The acquisition of complementary nadiral images reduced the error. Additionally, the sharp edges of the specimen show a negative error pattern (blue colors) related to the difficulties of the automatic matching process in this area where the useful surface is very limited.

**Figure 10:**
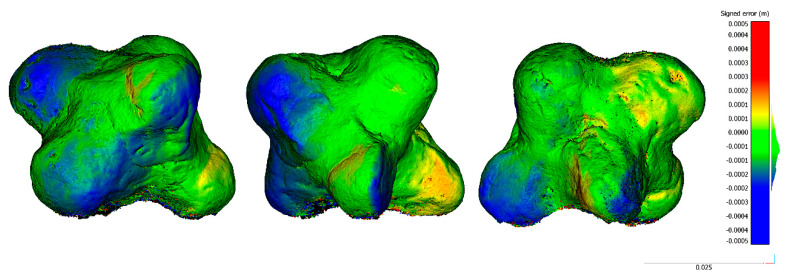
Different views of the spatial distribution of discrepancies between zoom lens reconstruction versus macro lenses for the specimen S1 (separator): front view (**left**); right side view (**center**); and back view (**right**).

**Figure 11:**
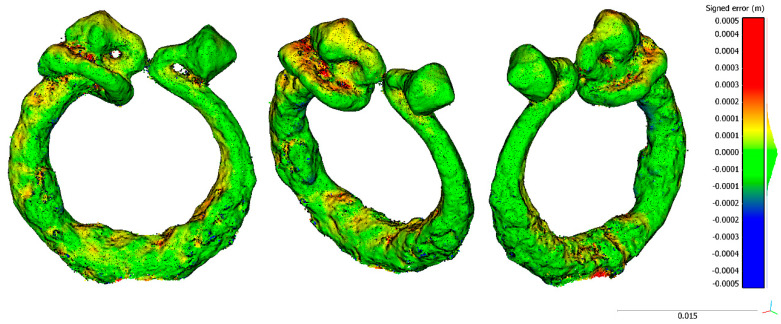
Different views of the spatial distribution of discrepancies between zoom lens reconstruction versus macro lenses for the specimen S2 (brooch): front view (**left**); front isometric view (**center**); and back isometric view (**right**).

Regarding specimens 1 and 2, as shown in Figure 10 and Figure 11, there are no significant error distributions. For specimen 1, in one of the laterals of the ends, there is a systematic negative discrepancy that could be related to the central tendency’s bias stated in Table 8. Regarding specimen 2, no bias was seen as expected by the median value compatible with zero (Table 8). The only significative discrepancies were in the top part, which could be caused by the challenging point of view for data acquisition.

Finally, the error increase from IPR 95% to IPR 99% (Table 9) was caused by the outliers of the manual cleaning of the rotating base and reusable adhesive putty. Therefore, the outliers should be taken into account in the evaluation of the photogrammetric configuration.

The lower spatial resolution and image definition of the zoom lenses would affect the GCP identification and therefore change the final 3D reconstruction. Since an ICP was applied, the rotation changes were dismissed in the analysis, remaining only shape deformation due to the error propagated by the GCPs. For all cases, the number of images and camera orientation was the same (therefore the baseline-to-depth ratio) and the lighting condition was controlled by the lightbox, which was the only significant error source is the employed lens. Regarding the GCP definition with the ACMM, the precision provided by this metrological instrument was higher than the photogrammetric reconstruction, thus it can be considered negligible. Remember that in both cases (Section 3.2) they were pre-calibrated independently.

## 4. Conclusions

In the present article, a new automatic protocol for the photogrammetric data acquisition is presented and evaluated. This protocol allows us to capture the images in a convergent path at equal angular intervals around a specimen. This configuration allows us to implement the data acquisition protocol of reconstructed small size archeological objects even if the operator is not an expert in photogrammetry, as is the case in an interdisciplinary field like archaeology. Furthermore, the images acquired using this protocol fully cover the geometry of the specimen without manually repositioning the camera, while providing an adequate dataset for the photogrammetric process using open [34,35,36,37] or commercial software [39]. The widely extended commercial application of Metashape [39] was chosen for this research due to its popularity and reduced complexity for final users (non-experts in photogrammetry). Both were chosen for their intuitive software interfaces and available documentation about use. The present approach is significant for three main reasons: aids in the reconstruction of small archaeological parts for documentation purposes [52]; helps assemble dismantled heritage elements and/or missing parts [29]; and generates didactical models for the acquisition of competences in an e-learning context [53] and can be included in products like augmented or virtual reality (AR/VR) applications for awareness-raising [6].

In this research, the variables that impact the photogrammetric reconstruction process was established as independent (e.g., luminosity, spatial reference system, specimen position, camera path, and photogrammetric reconstruction parameters). Only lens and calibration process were modified for the different experiments performed. Dense point clouds are generated for each case and there were control point errors for the pre-calibrated configurations between 0.072 and 0.204 mm. In this manner, a comparison between the point clouds obtained for the two lenses (macro and zoom) and the two calibration processes (pre-calibration and self-calibration) were implemented using a robust statistical analysis technique. Results show that the use of a non-macro lens does not substantially affect the geometric accuracy of the final 3D point cloud. However, when using a macro lens, the 3D model obtained is denser and can better reflect better small details of the geometry due to the smaller object sample distance (or GSD). In this regard, it should be noted that for the use of a macro lens, the establishment of an adequate depth of field allows for a proper focus of the entire object, which is a critical aspect that may not be easily solved by users without macro-photography experience. Therefore, and since the conventional zoom lens provided compatible results in terms of geometric error, they are more versatile and adequate for final users. Furthermore, as shown in the experimental results, a previous initial camera pre-calibration does not significantly improve the results for either lens, possibly due to the automated image acquisition, thanks to the robotic device. Moreover, the calibrated GCPs of the platform assure the metric quality of the 3D point cloud.

The results of the present research are expected to advise heritage specialists, which are non-experts in photogrammetry, about the data acquisition, lens selection, and modelling of small archaeological samples. With regard to future perspectives, there will be more test lenses and cameras with different sensor resolutions and specifications that use new comparison/validation techniques, which will thus increase the scope of the present work.

## Figures and Tables

**Table 1:** Main details about the archeological objects chosen for the research.

Archaeological Object	Approximate Age	Material	Archaeological Relevance
Specimen 1	I–II century A.D.	Backed clay	Separator. This piece was used in the clay ovens to separate the pieces. Its presence means the detection of ceramic activity.
Specimen 2	Roman chronology	Copper	Roman brooch (*fibula*). This object was used to join or fasten clothing. It indicates the habits and clothing of Roman age.
Specimen 3	2500 B.C.	Silex	Racloir. Flint tool made by prehistorical civilizations. Tool used for scraping, but it could be also used as a knife.

**Table 2:** Technical specifications of the photogrammetric system. CMOS: Complementary Meta Oxide Semiconductor.

**Camera Parameters**	**Canon EOS 77D**	
Sensor type	CMOS	
Sensor size	22.3 × 14.9 mm (APS C)	
Pixel size	0.0037 mm	
Image size	6000 × 4000 pixels	
Effective pixels	24.2 Mp	
**Lens Parameters**	**Conventional**	**Macro**
Principal distance	18–55 mm	60 mm
Diagonal field of view	73.4°/27.4°	25.2°
Aperture	f3.5/5.6–f22/38	f2.8–f32
Closer focused distance	0.250 m	0.20 m

**Table 3:** Technical specifications of metrological 7-axis arm contact measurement, hexagon absolute arm 7325SI.

Parameter	Value
Measuring range	2.5 m
Probing point repeatability	±0.049 mm
Probing volumetric accuracy	±0.069 mm
Scanning system accuracy	±0.084 mm

**Table 4:** Results for the bundle adjustment for the pre-calibrated configurations.

Specimen	Control Point Error	Check Point Error
S1—Macro	±0.150	±0.753
S1—Zoom	±0.084	±0.729
S2—Macro	±0.204	±0.837
S2—Zoom	±0.115	±0.854
S3—Macro	±0.109	±0.804
S3—Zoom	±0.072	±0.748

**Table 5:** Summary of the reconstruction process.

Specimen	Number of Images	Number of Points	Mean Spatial Resolution (mm)
S1—Separator	87	15,702,877 (macro)/2,909,894 (zoom)	0.02 ± 0.002 (macro)/0.04 ± 0.005 (zoom)
S2—Brooch	87	4,061,666 (macro)/503,757 (zoom)	0.02 ± 0.002 (macro)/0.05 ± 0.006 (zoom)
S3—Racloir	87	5,700,198 (macro)/2,202,236 (zoom)	0.02 ± 0.002 (macro)/0.03 ± 0.004 (zoom)

**Table 6:** Statistical analysis of the signed discrepancies. Units: millimeters.

Specimen	Mean	Std.	Median	NMAD	Sqrt (BWMV)	P 2.5%	P 5%	Q 25%	Q 75%	P 95%	P 97.5%
S1—Macro	0.00	±0.09	0.00	0.00	±0.01	−0.02	−0.01	0.00	0.00	0.01	0.02
S1—Zoom	0.00	±0.06	0.00	0.00	±0.00	−0.01	−0.01	0.00	0.00	0.01	0.01
S2—Macro	0.00	±0.08	0.00	0.00	±0.01	−0.02	−0.01	0.00	0.00	0.02	0.02
S2—Zoom	0.00	±0.04	0.00	0.01	±0.01	−0.02	−0.02	−0.01	0.01	0.02	0.02
S3—Macro	0.00	±0.10	−0.05	0.00	±0.00	−0.01	−0.01	0.00	0.00	0.01	0.01
S3—Zoom	0.00	±0.07	−0.05	0.00	±0.00	−0.01	−0.01	0.00	0.00	0.01	0.01

**Table 7:** Robust interpercentile ranges (IPR) and Gaussian confidence intervals (CI) for the pre-calibration/self-calibration comparison. Units: millimeters.

Specimen	Robust					Gaussian	
	IQR	IPR 68.27%	IPR 90%	IPR 95%	IPR 99%	CI 95%	CI 99%
S1—Macro	0.01	0.01	0.02	0.04	0.26	0.35	0.46
S1—Zoom	0.00	0.01	0.01	0.02	0.12	0.24	0.31
S2—Macro	0.01	0.01	0.03	0.04	0.37	0.31	0.41
S2—Zoom	0.01	0.02	0.03	0.04	0.09	0.16	0.21
S3—Macro	0.00	0.00	0.01	0.03	0.24	0.39	0.52
S3—Zoom	0.00	0.00	0.01	0.02	0.10	0.27	0.36

**Table 8:** Statistical analysis of the signed discrepancies. Units: millimeters.

Specimen	Mean	Std.	Median	NMAD	Sqrt (BWMV)	P 2.5%	P 5%	Q 25%	Q 75%	P 95%	P 97.5%
S1	−0.08	±0.19	−0.08	0.09	±0.09	−0.24	−0.21	−0.14	−0.02	0.07	0.10
S2	0.00	±0.17	0.00	0.07	±0.07	−0.15	−0.11	−0.04	0.05	0.13	0.18
S3	−0.05	±0.28	−0.05	0.07	±0.10	−0.52	−0.27	−0.10	0.00	0.23	0.32

**Table 9:** Robust interpercentile ranges (IPR) and Gaussian confidence intervals (CI) for the macro/zoom lens comparison. Units: millimeters.

Specimen	Robust					Gaussian	
	IQR	IPR 68.27%	IPR 90%	IPR 95%	IPR 99%	CI 95%	CI 99%
S1	0.12	0.18	0.28	0.34	1.10	0.74	0.98
S2	0.09	0.14	0.24	0.32	1.47	0.67	0.88
S3	0.10	0.20	0.50	0.84	2.07	1.10	1.44
